# Is mitral valve repair superior to replacement for chronic ischemic mitral regurgitation with left ventricular dysfunction?

**DOI:** 10.1186/1749-8090-5-107

**Published:** 2010-11-08

**Authors:** Zhibing Qiu, Xin Chen, Ming Xu, Yingshuo Jiang, Liqiong Xiao, LeLe Liu, Liming Wang

**Affiliations:** 1 Department of Cardiothoracic Surgery, Nanjing First Hospital affiliated to Nanjing Medical University, Nanjing Heart Institute, Nanjing, China

## Abstract

**Background:**

This study was undertaken to compare mitral valve repair and replacement as treatments for ischemic mitral regurgitation (IMR) with left ventricular dysfunction (LVD). Specifically, we sought to determine whether the choice of mitral valve procedure affected survival, and discover which patients were predicted to benefit from mitral valve repair and which from replacement.

**Methods:**

A total of 218 consecutive patients underwent either mitral valve repair (MVP, n = 112) or mitral valve replacement (MVR, n = 106). We retrospectively reviewed the clinical material, operation methods, echocardiography check during operation and follow-up. Patients details and follow-up outcomes were compared using multivariate and Kaplan-Meier analyses.

**Results:**

No statistical difference was found between the two groups in term of intraoperative data. Early mortality was 3.2% (MVP 2.7% and MVR 3.8%). At discharge, Left ventricular end-systolic and end-diastolic diameter and left ventricular ejection fraction (LVEF) were improved more in the MVP group than MVR group (P < 0.05), however, in follow-up no statistically significant difference was observed between the MVR and MVP group (P > 0.05). Follow-up mitral regurgitation grade was significantly improved in the MVR group compared with the MVP group (P < 0.05). The Kaplan-Meier survival estimates at 1, 3, and 5 years were simlar between MVP and MVR group. Logistic regression revealed poor survival was associated with old age(#75), preoperative renal insufficiency and low left ventricular ejection fraction (< 30%).

**Conclusion:**

Mitral valve repair is the procedure of choice in the majority of patients having surgery for severe ischemic mitral regurgitation with left ventricular dysfunction. Early results of MVP treatment seem to be satisfactory, but several lines of data indicate that mitral valve repair provided less long-term benefit than mitral valve replacement in the LVD patients.

## Background

Good-risk patients with ischemic mitral regurgitation (IMR) also benefit from mitral valve repair (MVP)compared with mitral valve replacement(MVR), with better early and late (5-year) survival, in part because of preservation of the subvalvar apparatus [1,2]. However, the presence of significant MR in the presence of left ventricular dysfunction (LVD) represents more advanced disease and is associated with a poor prognosis. There is discrepancy in the literature regarding the benefit of repair in IMR patients with LVD. In patients with LVD, the use of MVP instead of MVR has been questioned, with some centers reporting equivalent outcomes in select patients [3,4].

The purpose of this investigation was to review our experience of MVP versus MVR in LVD patients who underwent concomitant cardiac procedures to determine what differences, if any, exist in regard to morbidity and mortality. In addition, long-term mortality after repair and replacement in LVD patients was compared. Because selection of the valve repair or replacement procedure was not randomized, comparison required (1) to determine which patients were more likely to receive valve repair rather than replacement at this center, (2) to determine whether survival was better after mitral valve repair or replacement, (3) to discover which patients benefit from valve repair and which from replacement.

## Patients and methods

### Patient Selection in the Study

Ischemic mitral valve disease was classified from analysis of clinical information, operative reports, and echocardiograms. Thus all patients in this study had at least one previous myocardial infarction. Mitral regurgitation (MR) was defined as being ischemic in origin as evidenced by clinical data and echocardiographic findings. Mitral leaflets were normal, associated regional wall motion abnormality, and regurgitation was the result of completed MI, which is always present in the history of each patient [5,6]. Patients with functional IMR with Carpentier type IIIb and type I disease[6] were included in the study.

**Figure 1:**
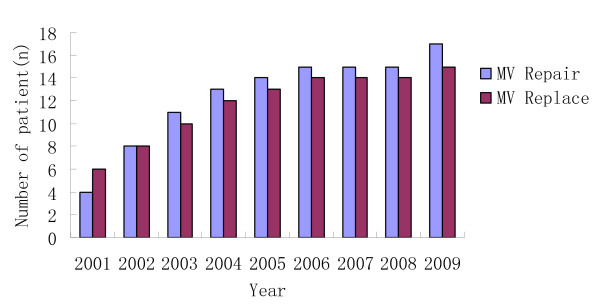
**Yearly distribution of patients**.

Data of 218 patients with significant chronic IMR who underwent CABG combined with mitral valve (MV) operations at a single institution from January 2001 through May 2009 were retrospectively analyzed. This reference group included patients who underwent MV repair (n = 112) and MV replacement (n = 106) during the same period. All patients had grade 3/4 or 4/4 MR on preoperative transthoracic echocardiography. Demographic and preoperative characteristics were shown in Table 1. Figure 1 showed trends in prevalences of both types of MV surgery by calendar year. No statistical difference was found between the two groups in term of the actual proportion of patients.

**Table 1:** Preoperative Data

	Mitral repair	Mitral replacement	*P *value
**Total number of patients**	112	106	

**Age>65 years**	75 (66.9%)	77 (72.6%)	**NS**
**Age range (years)**	70.6 ± 8.6	71.8 ± 10.8	**NS**
**Female**	40 (35.7%)	47 (44.3%)	**NS**
**Hypertension**	81(72.3%)	79(74.5%)	**NS**
**Diabetes mellitus**	33(29.5%)	34(32.1%)	**NS**
**Hyperlipidemia**	80(71.4%)	61(57.5%)	**0.032**
**Smoker**	76(67.9%)	81(76.4%)	**NS**
**COPD**	21(18.8%)	24(22.6%)	**NS**
**Pulmonary hypertension**	38(33.9%)	31(29.2%)	**NS**
**Chronic renal insufficiency**	8(7.1%)	6(5.7%)	**NS**
**Peripheral vascular**	4(3.6%)	3(2.8%)	**NS**
**Cerebrovascular accident**	3(2.7%)	2(1.9%)	**NS**
**Atrial fibrillation**	31(27.7%)	28(26.4%)	**NS**
**Previous MI(**<**30 days)**	12(10.7%)	10(9.4%)	**NS**
**Previous PCI**	70(62.5%)	45(42.5%)	**0.003**
**NYHA III-IV**	59(52.7%)	52(49.1%)	**NS**
**LVEF **<**30%**	22 (19.6%)	24 (22.6%)	**NS**
**Echocardiographic data**			
**LVEF (%)**	34.6 ± 5.5	35.1 ± 4.3	**NS**
**LVEDD (mm)**	66.29 ± 6.36	65.29 ± 6.36	**NS**
**LVESD (mm)**	50.21 ± 11.08	51.21 ± 11.08	**NS**
**LAD(mm)**	58.04 ± 17.26	57.86 ± 17.15	**NS**
**SPAP(mmHg)**	47.24 ± 14.31	48.01 ± 14.59	**NS**
**Left main disease >50%**	36(32.1%)	39(36.8%)	**NS**
**3-vessel disease**	91(81.3%)	88(83.0%)	**NS**
**Carpentier classification**[6]**, n (%)**			
**Ia**	40(35.7%)	35(33.1%)	**NS**
**IIIb**	72(64.3%)	71(66.9%)	**NS**
**Severe MR(+4), % (n)**	69(61.6%)	72(67.9%)	**NS**

COPD = chronic obstructive pulmonary disease; MI = myocardial infarction; mod = moderate; PCI = percutaneous intervention; NYHA = New York Heart Association; LVEF = left ventricular ejection fraction; LVEDD = left ventricular end-diastolic diameter; LVESD = left ventricular end-systolic diameter; LAD = left atrial diameter; SPAP = systolic pulmonary artery pressure; Left main disease = left main coronary stenosis; 3-vessel disease = triple coronary stenosis; MR = mitral regurgitation **NS **= **not significant;**

Exclusion criteria were mitral stenosis, aortic or tricuspid valve replacement, previous valve repair or replacement, emergency operation and non-ischemic mitral valve disease including degenerative, rheumatic, infective and congenital heart disease. The study protocol was approved by the institutional review committee of the Nanjing First Hospital. Patients gave informed consent.

### Surgical and Associated Procedures

All the patients had a standard monitoring, including a Swan Ganz catheter and transesophageal echocardiography. All procedures were performed through sternotomy by one surgeon (Dr. Xin Chen) during the study period. Patients were placed on cardiopulmonary bypass (CPB) using standard techniques. Dual venous cannulation was performed directly. Myocardial protection was achieved with antegrade and/or retrograde cold blood cardioplegia. When performed, coronary artery bypass graft (CABG) or atrial ablation procedure was done before the mitral procedure. Surgical approach was always transseptal. In case of incomplete vision, the incision was continued to reach the roof of the left atrium.

Myocardial revascularization was performed first. The mean number of bypassed vessels was 3.2 ± 1.0 in patients having MVP and 3.5 ± 1.2 in patients having MVR (*P *= 0.125). An internal thoracic artery graft was used for 93.8% of patients who underwent repair and 95.3% of those who underwent replacement (*P *= 0.620). Mitral annuloplasty always involved the posterior annulus and both commissures, and it was obtained by means of a suture annuloplasty. Multiple techniques were employed to achieve valve repair: leaflet resection, neo-chord insertion, chordal transfer and edge-to-edge approximation. When the MV was replaced, only a part of the anterior leaflet was excised to preserve the integrity of the subvalvular apparatus. Transesophageal echocardiography (TEE) was used routinely during intra-operative period. Before sternal closure, cold saline was injected to confirm competence of the repair and TEE was performed to confirm satisfactory MV function. Aortic cross-clamp time was 105 ± 42 minutes in the mitral valve repair group and 98 ± 39 minutes in the mitral valve replacement group (*P *= 0.158). Perioperative patient characteristics are given in Table 2.

**Table 2:** Operative Details and Associated Procedures

	Mitral repair (n = 112)	Mitral replacement (n = 106)	*P *value
**Valve repair techniquea**			
**Triangular resection**	60(53.6%)		
**Quadrangular resection**	36(32.1%)		
**Neochord insertion**	10(8.9%)		
**Chordal transfer**	3(2.7%)		
**Edge-to-edge repair**	3(2.7%)		
**Annuloplasty ring**	112(100%)		
**Valve replacement**			
**Hancock porcine**		46(43.4%)	
**Carpentier-Edwards pericardial**		20(18.9%)	
**St. Jude mechanical**		22(20.7%)	
**Carbomedic mechanical**		18(17.0%)	
**LV reconstruction**	7(6.3%)	6(5.7%)	0.854
**Atrial ablation/appendage ligation**	29(25.9%)	24(22.6%)	0.576
**Coronary artery bypass grafting**	112(100%)	106(100%)	1.000
**Number of bypassed vessels**	3.2 ± 1.0	3.5 ± 1.2	0.125
**internal thoracic artery graft**	105(93.8%)	101(95.3%)	0.620
**Cross-clamp time(min)**	105 ± 42	98 ± 39	0.158
**CPB time(min)**	136 ± 50	129 ± 41	0.424

At the end of the procedure, all patients electively received 5 μg·kg^-1^·min^-1 ^of dobutamine and either nitroglycerin or sodium nitroprusside according to arterial resistance. Other inotropic agents, as well as an intra-aortic balloon pump, were used when necessary.

### Echocardiography

All the patients had a preoperative transthoracic echocardiogram. The mitral annulus was identified as the leaflet hinge point, and its size was measured in the apical long axis, four- and two-chamber views at the end of systole; the mean value was considered. The distance between the points where the MV leaflets coapt and the mitral annulus plane was measured at end-systole in the four-chamber apical long axis view. Left ventricular end-systolic diameter (LVESD), left ventricular end-diastolic diameter (LVEDD), left atrial diameter (LAD) and systolic pulmonary artery pressure (SPAP) were measured from parasternal M-mode acquisitions, and left ventricular ejection fraction (LVEF) was calculated using the biplane Simpson method [7].

Preoperative and postoperative echocardiographic data were recorded. The presence and entity of MR were evaluated by using colored areas of jet regurgitation and jet-to-left atrium area ratios [8]. Based on echocardiography, MR severity was graded as no or trivial regurgitation (0), mild (1+), moderate (2+), moderate to severe (3+), or severe (4+). All patients had 3+ to 4+ before surgery (mean 3.59 ± 0.40+).

### Follow Up

Follow-up (FU)was achieved by direct telephone contact with the patient, family, primary care physician, or cardiologist. All living patients or their relatives were mailed a questionnaire that contained questions related to the patient's current health status, medication, cardiac death, and any cardiac events during follow-up. Two patients were lost to follow-up in MVP group, and three patients were lost in MVR group. The mean duration of follow-up was 48.1 ± 13.7 months (range, 2 to 96 months) and 50.2 ± 14.4 months (range, 3 to 98 months) in patients with MVP and MVR, respectively. At that moment, when possible, a transthoracic echocardiogram was performed by our cardiologists.

The primary end-points were to evaluate early and midterm survival, the New York Heart Association (NYHA) functional class and echocardiographic modifications of left ventricle and the presence of any grade of IMR. *Cardiac death *was defined as death cardiac related or sudden death; *cardiac event *as the occurrence of at least one of following event: acute myocardial infarction, surgical or interventional reoperation.

### Data Collection

Perioperative risk factors and demographics were determined from the database and supplemented by chart review. Postoperative data were collected from patients' hospital charts. Echocardiographic data were collected from patients' charts and hospital records. The data were supplemented by interviews with primary care physicians and cardiologists. Strategies for surgical revascularization and for choice of mitral prosthesis were at the discretion of the surgeon. Mortality data were obtained from chart review and review of death certificates.

### Statistical Analysis

Results are expressed as mean ± standard deviation unless otherwise indicated. Statistical analysis comparing two independent groups was performed with unpaired two-tailed Student's *t *test for the means or χ^2 ^test for categorical variables. Logistic regression was used to identify risk factors for survival. Kaplan-Meier survival curve estimates were used to compare actuarial survival rates between mitral repair and replacement in LVD patients. The SPSS 13.0 software (SPSS Inc, Chicago, IL) was used. Probability values less than 0.05 were considered significant.

Variables examined by logistic regression analysis in terms of risk factors of the surgical procedure included the following: age older than 65 years, preoperative chronic obstructive pulmonary disease, previous PCI, preoperative stroke or transient ischemic attack, left main disease, preoperative LVEF less than 30%, renal dysfunction (serum creatinine>2.0 mg/dl), Mitral valve repair and replacement [9].

## Results

### Baseline Characteristics

Table 1 summarizes the preoperative patient characteristics. All patients had symptomatic CAD, 10.1% had had a myocardial infarction within 30 days of the operation, and 50.9% had New York Heart Association class III or IV symptoms of heart failure. The two groups were similar in terms of age, gender, incidence of diabetes mellitus, baseline NYHA class, baseline LVEF, and number of vessel disease. The MVP group had significantly more patients with hyperlipidemia (MVP 71.4% versus MVR 57.5%, *P *= 0.032) and previous PCI (MVP 62.5% versus MVR 42.5%, *P *= 0.003) at baseline. This was not unexpected since the patients were not randomized and the decision whether to repair or replace the mitral valve was based at least in part on these characteristics.

### Intraoperative characteristics

The type of mitral repair is shown in Table 2. All MVP patients had ring annuloplasty and the median size of the annuloplasty ring used was 30 mm (range, 26 to 34 mm). Ten patients (8.9%) with neo-chord insertion, 3 patients (2.7%) with chordal transfer and 3 patients (2.7%) with edge-to-edge valvuloplasty were adopted in anterior leaflet prolapse. Among patients who had a mitral valve replacement, 94 (88.7%) had preservation of the posterior mitral leaflet with part excision of the anterior leaflet, and 12 (11.3%) had bileaflet preservation. Among patients undergone mitral valve replacement, 62.3% received bioprosthesis, and 37.7% received mechanical valves. No statistical difference was found between the two groups in term of intraoperative data, including CPB time, aortic cross-clamp time and number of bypass grafts (*p*>0.05).

### Perioperative morbidity and mortality

Postoperative data with duration of mechanical ventilation, ICU treatment, complications and hospital stay are listed in Table 3. Mean intensive care unit stay and mean hospital stay had no statistical difference between the two groups. In 49 patients (22.5%) intra-aortic balloon pump (IABP) was inserted, with 28 patients preoperative insertion and 21 postoperative insertion (MVP 20.5% versus MVR 24.5%, *P *= 0.480). Five patients (5%) required operative re-exploration because of bleeding (MVP 1.8% versus MVR 2.8%, *P *= 0.607). Seven patients needed readmission in the ICU for acute respiratory insufficiency(MVP 2.7% versus MVR 3.8%, *P *= 0.647). Furthermore, Table 3 demonstrates no difference between the two groups occurred in terms of acute myocardial infarction (0.89% in MVP, 0.94% in MVR, *P *= 0.969), cerebrovascular accident (1.8% in MVP, 2.8% in MVR, *P *= 0.607), low output syndrome (16.1% in MVP, 15.1% in MVR, *P *= 0.842), and Acute renal failure(4.5% in MVP, 3.8% in MVR, *P *= 0.798). No patients required reoperation after an initial mitral valve replacement. One patient needed to mitral valve replacement in the repair group, due to endocarditis.

**Table 3:** Perioperative datas

	Mitral repair(n = 112)	Mitral replacement(n = 106)	*P *value
**In-hospital(< 30 day) mortality**	3(2.7%)	4(3.8%)	0.647
**AMI**	1(0.89%)	1(0.94%)	0.969
**CVA**	2(1.8%)	3(2.8%)	0.607
**LOS**	18(16.1%)	16(15.1%)	0.842
**IABP support**	23(20.5%)	26(24.5%)	0.480
**Acute renal failure**	5(4.5%)	4(3.8%)	0.798
**Acute respiratory failure**	3(2.7%)	4(3.8%)	0.647
**Bleeding (mL/12 h)**	2(1.8%)	3(2.8%)	0.607
**Sepsis or endocarditis**	1(0.89%)	0	0.330
**ICU stay (h)**	3.6 ±0.8	3.9 ± 1.0	0.265
**In-hospital stay (d)**	18.0 ± 8.2	19.5 ± 9.1	0.313

AMI = acute myocardial infarction; CVA = cerebrovascular accident; LOS = low-output syndrome; IABP = intraaortic balloon pump; ICU = intensive care unit; MV = mitral valve; NS = not significant.

Seven patients died during the first 30 postoperative days: two died as a result of low output syndrome, and five were lost for non-cardiac causes (rupture of abdominal aneurysm, tracheal bleeding, and multi-organ failure as a result of bleeding). Early mortality was 3.2% (7 of 218 patients). Three of them had undergone MV repair (2.7%) and four had undergone MV replacement (3.8%; *P *= 0.647). Logistic regression did not show that mitral repair or replacement would be significant risk factors for early mortality according to the risk ratio for survival (*p*>0.05).

### Follow up mortality and outcomes

Mean follow-up of the survivors was 49.6 ± 12.5 months, with 18 patients (8.5%) died, 6 of cardiac causes (heart failure in 3, sudden death in 1, and acute MI in 2) and 12 died of non-cardiac causes (cerebrovascular accident in 4, septicemia in 3, car accident in 2, acute respiratory failure in 2, and renal failure in 1). Ten of them (9.2%) had undergone MV repair and eight had undergone MV replacement (7.8%). The cumulative survival rate for both groups, including in-hospital mortality, is shown in Figure 1. And no statistically significant difference was found between the two groups.

At discharge, NYHA class in the MVP group improved from 2.9 ± 1.0 to 1.5 ± 0.4, but in the MVR group it improved from 2.8 ±0.7 to 2.3 ±0.7 (MVP versus MVR, *p *< 0.05, Table 4). At the last follow-up, NYHA class III or greater was present in 21 (19.6%) patients in the MVP group and in 11 (11.1%) patients in the MVR group (MVP versus MVR, *p *< 0.05). There was no hemorrhaging, thromboembolic complications, or residual leakage or stenosis during follow-up.

**Table 4:** Follow-up Clinical and Echocardiographic Results

	Mitral repair			Mitral replacement		
	
	Preoperative (n = 112)	At discharge (n = 109)	Follow-up (n = 107)	Preoperative (n = 106)	At discharge (n = 102)	Follow-up (n = 99)
**Follow-up duration (month)**	48.1 ± 13.7			50.2 ± 14.4		
**NYHA class (Mean ± SD)**	2.9 ± 1.0	1.5 ± 0.4 ^a^	1.9 ± 0.5 ^a^	2.8 ± 1.0	2.3 ± 0.7 ^a b^	1.6 ± 0.4 ^a^

**NYHA class III or greater(n)**	59(52.7%)	10(9.2%) ^a^	21(19.6%) ^a^	52(49.1%)	23(22.5%) ^a^	11(11.1%) ^ac^

**LVEDD(mm)**	66.29 ± 6.36	54.01 ± 5.15 ^a^	49.01 ± 4.57 ^a^	65.35 ± 6.29	62.14 ± 5.06 ^a b^	50.22 ± 4.35 ^a^
**LVESD(mm)**	50.21 ± 11.08	43.09 ± 8.54 ^a^	39.12 ± 7.52*	51.12 ± 11.53	48.34 ± 8.02 ^a b^	40.06 ± 7.76 ^a^
**LAD(mm)**	58.04 ± 17.26	53.31 ± 15.03 ^a^	48.32 ± 9.34 ^a^	57.86 ± 17.15	54.02 ± 15.28 ^a^	40.21 ± 8.05 ^ac^
**LVEF (%)**	34.6 ± 5.5	45.3 ± 4.3 ^a^	54.2 ± 3.1 ^a^	35.1 ± 4.3	40.2 ± 4.9 ^a b^	55.1 ± 3.6 ^a^
**SPAP(mmHg)**	47.24 ± 14.31	40.43 ± 10.52 ^a^	37.07 ± 8.26 ^a^	48.01 ± 14.59	40.05 ± 10.12 ^a^	31.24 ± 7.13 ^ac^
**Grade of MR (Mean ± SD)**	3.57 ± 0.38	0.95 ± 0.36 ^a^	1.30 ± 0.65 ^a^	3.42 ± 0.35	0.15 ± 0.05 ^a^	0.40 ± 0.10 ^ac^
**Carpentier classification**[6]**, n (%)**						
**Ia MR**	40(35.7%)	40(36.7%)	38(35.5%)	35(33.1%)	33(32.4%)	32(32.3%)
**IIIb MR**	72 (64.3%)	69(63.3%)	69(64.5%)	71(66.9%)	69(67.6%)	67(67.7%)

Compares with Preoperative ^a ^*p *< 0.05; significantly MVR versus MVP group at discharge ^b ^*p *< 0.05; MVR versus MVP group in follow-up ^c^*p *< 0.05.

NYHA = New York Heart Association; LVEDD = left ventricular end-diastolic diameter; LVESD = left ventricular end-systolic diameter; LAD = left atrial diameter; LVEF = left ventricular ejection fraction; SPAP = systolic pulmonary artery pressure; MR = mitral regurgitation; SD = standard deviation.;

### Follow up echocardiographic evaluation

The last known echocardiogram was found in 98.2% (107 of 109) of MVP group patients and 97.1% (99 of 102) of MVR group patients in follow up. At discharge, LVEDD (*p *< 0.05), LVESD (*p *< 0.05) and LVEF (*p *< 0.05) were more decreased in the MVP group versus that seen in the MVR group. However, follow-up left ventricular reversal remodeling measured by change in LVEDD (*p *< 0.05), LVESD (*p *< 0.05), and LVEF (*p *< 0.05) was significantly observed in the MVR group with respect to baseline values, but no statistically significant difference in left ventricular reversal remodeling was observed in the MVP group (*p*>0.05). In the MVR group we found an improvement in SPAP at follow-up with respect to patients in the MVP group (*p *< 0.05) and to baseline values (*p *< 0.05). Follow-up LAD changed from 57.86 ± 17.15 to 40.21 ± 9.05 mm in the MVR group and from 58.04 ± 17.26 to 48.32 ± 9.34 mm (*p *< 0.05) in the MVP group. Follow-up MR grade was significantly improved in the MVR group compared with the MVP group (*p *< 0.05). Data are presented in Table 4.

### Is Survival Better After Mitral Valve Repair Than After Replacement?

**Figure 2:**
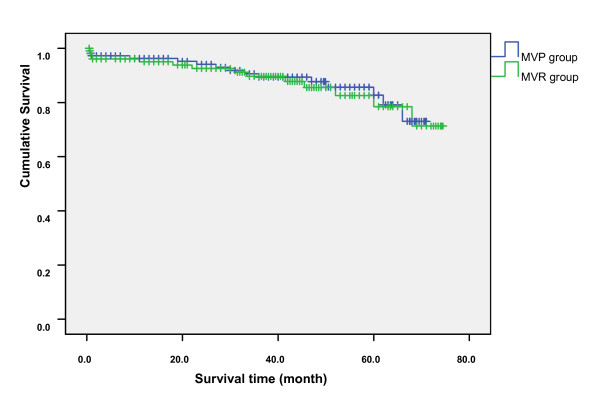
**Long-term survival with mitral valve repair (blue line) versus replacement (green line) in LVD patients**.

After accounting for postoperative deaths, survival between repair and replacement in LVD patients was similar (P > 0.05). During the follow-up period, no patient in the MVR group required reoperation for his or her MV. Kaplan-Meier survival estimates at 1, 3, and 5 years were 0.96, 0.89, and 0.73 in MVP group, and 0.95, 0.88, and 0.71 in MVR group (Figure 2). Overall survival distributions was equivalent in LVD patients undergoing repair versus replacement (P > 0.05).

Multivariate analysis on all patients was performed to account for confounding factors and included clinically relevant risk factors (Table 5). After logistic regression, independent predictors of decreased survival was associated with age of 75 years or older (odds ratio, 1.89; *p *< 0.05) and highly associated with preoperative renal insufficiency (odds ratio, 3.27; *p *< 0.01) and LVEF < 30% (odds ratio, 2.41; *p *< 0.01). Preoperative arrhythmia, MV replacement, concomitant operations, reoperation, and left main disease were not found to be significant prognostic factors.

**Table 5:** Prognostic Factors for Survival ^a ^After Mitral surgery for Ischemic Mitral Regurgitation

*Prognostic Factors*	***Survival***^***a***^		
	
	OR	95% CI	*p *Value
Age > 65 years	1.89	1.01-2.86	0.012
Female	1.29	1.14-1.48	0.182
Preoperative renal insufficiency	3.27	1.52-4.64	0.003
COPD	0.99	0.81-1.23	0.221
Preoperative PCI	1.22	0.83-1.75	0.454
Previous stroke	1.35	0.67-2.81	0.323
left main disease	0.84	0.52-1.25	0.434
LVEF < 30%	2.41	1.30-3.15	0.002
Mitral valve replacement	1.27	0.78-2.14	0.630
Mitral valve repair	0.92	0.45-1.95	0.270
Reoperative procedure	1.00	0.87-1.17	0.945

a Multivariate Cox regression analysis of patients who survived >30 days. CI = confidence interval; OR = odds ratio; COPD = chronic obstructive pulmonary disease; LVEF = left ventricular ejection fraction.

## Discussion

Although the results of mitral repair for IMR have improved over the last 20 years, until recently, surgical correction of IMR in the setting of severe left ventricular dysfunction was considered anathema. Bolling and colleagues [10] demonstrated that this approach was feasible and could be conducted with reasonably low morbidity, using an undersized annuloplasty repair effectively corrects MR in heart failure patients. Romano and Bolling [11] have reported their observational experience in more than 200 patients with severe MR and left ventricular ejection fraction < 0.20) with mitral valve repair. The 1-, 2-, and 5-year actuarial survival rates were 82%, 71%, and 52%, respectively. New York Heart Association class improved for all patients and at the 24-month follow-up; However, patients in NYHA class IV with extreme left ventricular dysfunction have poor survival, regardless of mitral valve procedure, and present a contemporary surgical challenge [12]. The central questions pertinent to the treatment of ischemic mitral insufficiency by repair or replacement techniques include effectiveness, appropriateness, and long-term benefits.

### For Which Patients Is Repair or Replacement Appropriate?

Although the applicability of MVP is easily appreciated in the subset of patients with chronic ischemia and annular dilation, it is noteworthy that 21.1% (46/218)of the repairs in our series were done in cases of severe LVD (LVEF < 30%). These cases are challenging to the surgeon because evaluation of the damage to the subvalvular apparatus may be difficult. Not only does structural damage (ruptured chordae or papillary muscle) need to be readily discerned, but subtle, ongoing pathologic processes of the subvalvular apparatus must also be accurately appraised. The few patients with valve reconstruction who required reoperation did so within a short period after the original operation. Although there was a trend toward further re-intervention in the patients with valve repair, this difference can be attributed mainly to the learning curve associated with recognizing the extent of reconstruction in IMR. It is important to note that in our series there were no late valve-related deaths among patients undergoing further mitral valve surgery.

Surgical techniques for mitral valve repair in patients with ischemic mitral regurgitation have been described by others [13,14]. Functional ischemic mitral regurgitation was repaired by annuloplasty alone. We prefer to use an undersized annuloplasty, and most of patients who underwent mitral valve repair had an annuloplasty that was 30 mm or smaller. Others have also reported excellent results with an undersized annuloplasty for functional ischemic mitral regurgitation [15]. In our institution, we have adopted Gore-Tex neo-chord, chordal transfer or edge-to-edge valvuloplasty to use in anterior leaflet prolapse without excess tissue.

However, the possibility of allowing both leaflets to coapt depends on the ability of the anterior leaflet to move toward the annulus and to reach the posterior one. If this movement is insufficient, the mitral leaflets never coapt no matter how much the posterior annulus is reduced. For this reason, for each patient, we evaluate the depth of the anterior leaflet during systole. According to our experience this value is crucial for deciding whether to repair (if 10 mm or less) or to replace (if more than 10 mm) the MV, which corresponds with results of earlier reports [16]. Moreover, the 5-year results appear to be similar to the results in patients undergoing MV repair and replacement, although it is likely that curves can diverge significantly with a longer follow-up and a greater number of patients. This finding focuses on preventing MR recurrence (or reducing it as much as possible), which is the main target of MV surgery for IMR.

Earlier reports have shown that use of preoperative IABP therapy can reduce myocardial ischemia and therefore improve outcome in high-risk patients undergone CABG with the use of CPB [17]. Recent reports have indicated that pre- and perioperative IABP therapy facilitates manipulation of the heart with maintained hemodynamic stability and with reduced myocardial oxygen demand in high-risk patients undergoing CABG surgery [18]. In the study, there were 19 patients preoperatively inserted IABP and 10 patients postoperatively IABP therapy.

### Is Mitral Reconstruction an Effective Treatment Option?

Patients with IMR and LVD have an unfavorable prognosis, with poor survival relative to patients with other causes of mitral dysfunction [19,20]. It is therefore important to determine which factors influence early and late survival for risk stratification and alteration of surgical approach that might improve survival. We documented several risk factors for early and late death after surgical treatment of ischemic mitral regurgitation. These included such general factors as older age, advanced NYHA functional class, severe left ventricular dysfunction, and preoperative renal dysfunction.

An attempt to preserve the native MV apparatus to maintain the normal shape, volume, and function of the LV by reparative surgery is always preferred to valve replacement. If successful, the risk of long-term anticoagulation and prosthetic valve complications are also avoided. Mitral valve repair leads to improved survival as compared to MV replacement. Mitral valve replacement with preservation of the subvalvular apparatus gives significantly better results as compared to MV replacement without preservation. Resection of the entire subvalvular apparatus should almost never be contemplated except in severely calcified valves.

Therefore, recent studies have reported that early mortality of MVR is reducing, and is becoming similar to MVP for patients with IMR and similar EF [21,22]. Our study shows that in a population of high risk patients it is possible to achieve an acceptable and similar early mortality between MVP and MVR group.

### Impact of MV Repair and Replacement on Ischemic MR and LV Remodeling

In functional ischemic MR, the MV is structurally normal and MR is caused by dysfunction of the LV, resulting in incomplete leaflet closure [23]. We found that in patients with functional recovery of the LV, the severity of MR and LV size were significantly decreased after surgery, because revascularization may improve LV dysfunction and geometry, restoring valvular coaptation and thereby improving ischemic MR. At discharge, LVEDD, LVESD and LVEF were more decreased in the MVP group versus that seen in the MVR group. However, in follow-up reversal in left ventricular remodeling measured by change in LVEDD, LVESD, and LVEF was significantly observed in the MVR group with respect to baseline values, but no statistically significant difference in left ventricular reversal remodeling was observed in the MVP group.

LV reverse remodeling had been observed after restrictive mitral annuloplasty, whereas the grade of MR occurred higher after MVP than MVR, indicating that LV remodeling might be a progressive ventricular problem that cannot be treated by annuloplasty. In an experimental ovine model, prophylactic ventricular restraint attenuated adverse remodeling and reduced ischemic MR severity, whereas prevention of MR by ring annuloplasty did not influence remodeling [24,25].

Previous clinical studies have compared the results of MV repair against those following MV replacement and have concluded that preservation of the annular-chordal-papillary muscle continuity results in maintenance of LV function and geometry, leading to better patient outcome [21,26]. However, we could not observe a difference in outcome between MV repair and replacement. One reason could be the preservation of the mitral valve apparatus despite MV replacement. But we think that chordal sparing mitral valve replacement is not a better way to treat IMR because of the need for anticoagulation for mechanical prosthesis in mitral position and inevitable degeneration of bioprosthesis.

### What Are the Long-term Benefits?

Recent reports have successfully compared late results with repair versus replacement for ischemic MR in a statistically controlled fashion [21,27]. Both studies suggested that MV repair may be better in low-risk patients, but as expected the patient populations were diverse. One study concluded that 70% of patients with ischemic MR benefit from repair over replacement, but in the high-risk setting, or with complex regurgitant jets, survival were similar with both techniques [28]. In the current report, the 5-year survival among the patients with mitral repair and replacement in this series ranged from approximately 71% to 73%. Gillinov and associates [2] had 30-day mortality of 13% and, in the lower-risk group, a 5-year survival of 58% after MV repair and of 36% after MV replacement; in the higher risk group, survival after either repair or replacement was similarly poor. The authors concluded that even though most patients with IMR benefit from MV repair, in the most-complex, high-risk settings, survival after either repair or replacement is similar. And survival is related to the degree of impairment of LV, so this may be the cause of lack of difference in survival between repair and replacement.

As recurrent MR after ring anuloplasty relates to LV remodeling, approaches that also alleviate ventricular remodeling could potentially be part of a more comprehensive and effective management strategy for IMR [29]. Therefore, MV replacement with intact subvalvular apparatus should be considered in patients with chronic IMR who have multiple comorbidities, complex regurgitant jets, or severe tethering of both mitral valve leaflets.

### Limitations

This is a single-institution retrospective review, a limitation to most of the literature comparing MV repair to replacement. As such, there may be a selection bias for valves that are able to be repaired. The repairability of a valve including the complexity of valve disease and degree of annular calcification is difficult to assess by reviewing operative notes of patients who underwent mitral replacement and is a clear limitation to the potential bias in our report. A standardized intraoperative assessment model would be helpful in this and future multicenter studies.

Finally, patients with intermittent ischemic mitral regurgitation treated by coronary revascularization alone were not included in this analysis. Despite the limitations, this study reaffirms the grave prognosis associated with significant IMR and identifies predictors of early and late death.

## Conclusion

The efficacy of adding mitral valve repair to coronary artery bypass grafting is well demonstrated by the improvement of New York Heart Association functional class and percentage of left ventricular ejection fraction and by the decrease of left ventricular end-diastolic diameter, left ventricular end-systolic diameter, pulmonary artery pressure, and left atrial size. Early results seem to be satisfactory, even when most of these patients are in preoperative congestive heart failure.

However, there is a perception that MV repair does not provide long-term benefit in the most IMR patients with LVD. When mitral valve repair is performed, a formal annuloplasty should be used, and it is a beneficial effect of preoperative IABP treatment in IMR patients with LVD undergone MV surgery. At this end of the spectrum, survival and freedom from mitral valve reoperation were similar after repair and replacement, whereas the grade of recurrent MR occurred higher after MVP than MVR.

## Competing interests

The authors declare that they have no competing interests.

## Authors' contributions

QZB and CX had helped with design of the study, data interpretation and in writing of the paper. XM has made the statistical analysis and took part in the writing process. QZB also took part in the correction of the manuscript according to the reviewers' suggestions. JYS and WLM had helped in gathering patient information and performed graphic measurements. XLQ and LLL performed graphics and tables and added comments to the paper. All authors read and approved the final manuscript.
